# Cross-linking mass spectrometry identifies new interfaces of Augmin required to localise the γ-tubulin ring complex to the mitotic spindle

**DOI:** 10.1242/bio.022905

**Published:** 2017-03-28

**Authors:** Jack W. C. Chen, Zhuo A. Chen, Kacper B. Rogala, Jeremy Metz, Charlotte M. Deane, Juri Rappsilber, James G. Wakefield

**Affiliations:** 1 Department of Biosciences, College of Life and Environmental Sciences, University of Exeter, Stocker Road, Exeter EX4 4QD, UK; 2 Wellcome Trust Centre for Cell Biology, University of Edinburgh, Max Born Crescent, Edinburgh EH9 3BF, UK; 3 Department of Statistics, University of Oxford, South Parks Road, Oxford OX1 3TG, UK; 4 Chair of Bioanalytics, Institute of Biotechnology, Technische Universität Berlin, 13355 Berlin, Germany

**Keywords:** γ-TuRC, Augmin, *Drosophila*, Microtubule, Mitosis, Spindle

## Abstract

The hetero-octameric protein complex, Augmin, recruits γ-Tubulin ring complex (γ-TuRC) to pre-existing microtubules (MTs) to generate branched MTs during mitosis, facilitating robust spindle assembly. However, despite a recent partial reconstitution of the human Augmin complex *in vitro*, the molecular basis of this recruitment remains unclear. Here, we used immuno-affinity purification of *in vivo* Augmin from *Drosophila* and cross-linking/mass spectrometry to identify distance restraints between residues within the eight Augmin subunits in the absence of any other structural information. The results allowed us to predict potential interfaces between Augmin and γ-TuRC. We tested these predictions biochemically and in the *Drosophila* embryo, demonstrating that specific regions of the Augmin subunits, Dgt3, Dgt5 and Dgt6 all directly bind the γ-TuRC protein, Dgp71WD, and are required for the accumulation of γ-TuRC, but not Augmin, to the mitotic spindle. This study therefore substantially increases our understanding of the molecular mechanisms underpinning MT-dependent MT nucleation.

## Abstract (precis)

**Summary:** A map of subunit positional restrictions in *Drosophila* Augmin shows that Dgt3, Dgt5 and Dgt6 are all required to localise gamma-TuRC to mitotic spindles.

## INTRODUCTION

Since its discovery in *Drosophila* (Goshima et al., 2007, 2008; Hughes et al., 2008), the Augmin complex has radically changed our understanding of microtubule (MT) generation during mitosis. Augmin amplifies MT number during mitosis and without it, the density of MTs within the mitotic spindle is dramatically reduced, such that chromosome alignment and mitotic progression are perturbed (Uehara et al., 2009; Lawo et al., 2009; Wainman et al., 2009; Meireles et al., 2009; Bucciarelli et al., 2009; Ho et al., 2011; Petry et al., 2011; Hotta et al., 2012; Hayward et al., 2014). Each of the eight proteins that constitute Augmin localise to MTs (Goshima et al., 2007; Hughes et al., 2008) and, in humans, the HAUS6 (FAM29A) subunit has been shown to associate with NEDD1, part of the MT nucleating γ-Tubulin ring complex (γ-TuRC) (Zhu et al., 2008; Teixidó-Travesa et al., 2010). Moreover, removal of *Drosophila* Augmin, through RNAi, mutation or immuno-depletion, removes the fraction of γ-TuRC normally present on the spindle, without affecting centrosomal levels (Goshima et al., 2007, 2008; Wainman et al., 2009); a phenotype similar to that seen upon loss of the NEDD1 homologue, Dgp71WD (Reschen et al., 2012). The current model is therefore that Augmin acts as a molecular linker between an existing MT and a γ-TuRC, allowing the nucleation of new MTs from the walls of pre-existing ones; a hypothesis supported by observations in *Drosophila*, *Xenopus* and plants (Kamasaki et al., 2013; Petry et al., 2013; Liu et al., 2014). However, the relationship between Augmin structure and function is still poorly understood, due both to its multi-subunit complexity and to the very limited homology of Augmin between species; only four of the eight Augmin subunits are conserved between humans and invertebrates at the primary structure level (Dgt6/HAUS6, Dgt4/HICE1/HAUS8, Dgt3/HAUS3 and Dgt5/HAUS5); and even within these, the homology is restricted (Uehara et al., 2009; Duncan and Wakefield, 2011). Although a recent *in vitro* partial reconstitution of human Augmin identified direct interactions between specific subunits (Hsia et al., 2014), it also highlighted the limitations of a ‘bottom-up’ *in vitro* reconstitution approach to understanding Augmin function; and the structural integrity of the full complex and its relationship to mitotic function remains unclear.

Here we took an alternative, *in vivo*-driven approach; using cross-linking/mass spectrometry (CLMS) (Rappsilber, 2011) of Augmin, purified directly and endogenously from *Drosophila* embryos, to predict the orientation of the subunits within the complex and the likely interfaces that facilitate interaction with γ-TuRC. Validation of these predictions using both direct protein-protein assays and through injecting domains of subunits into *Drosophila* embryos, identified multiple subunit interfaces required to recruit γ-TuRC to the mitotic spindle. This study therefore highlights both the complexity of regulating MT-dependent MT nucleation in the cell and the predictive power of CLMS.

## RESULTS AND DISCUSSION

**Fig. 1.**
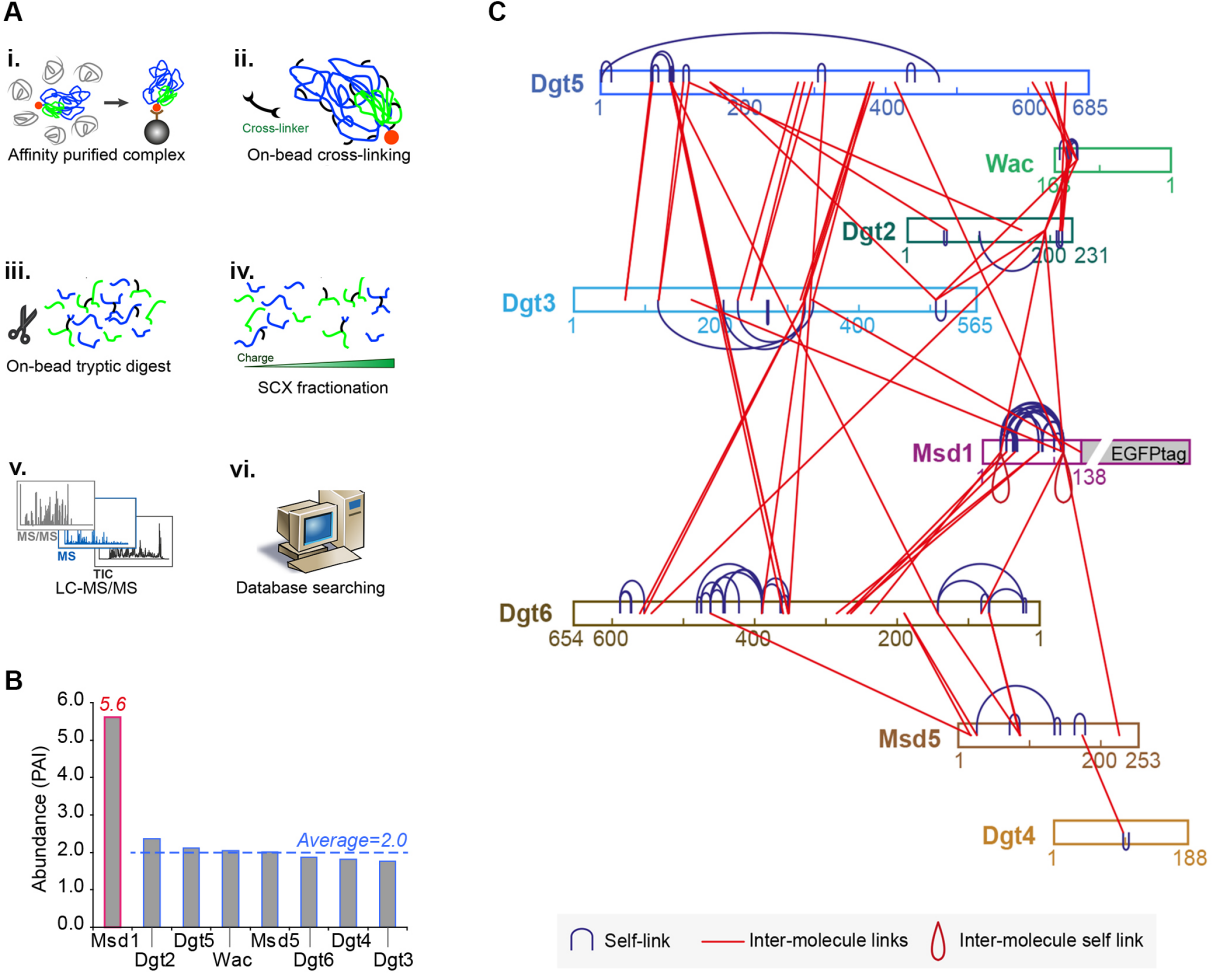
**Cross-linking mass spectrometry (CLMS) of Augmin.** (A) Methodology used for CLMS of Augmin isolated from extracts of *Drosophila* embryos expressing GFP-Msd1. (B) Relative abundance (mean area) of each Augmin subunit, as identified by LC-MS/MS. (C) Cross-links within and between Augmin subunits, as identified by CLMS. Subunits are shown as coloured bars and labelled according to amino acid position (N- to C- terminal).

We have previously shown that transgenic flies expressing a GFP-tagged variant of the *Drosophila* Augmin subunit, Msd1, rescue the female sterility and mitotic spindle defects associated with a mutation in the *msd1* gene (Wainman et al., 2009). We subjected extracts from syncytial *Drosophila* embryos expressing Msd1-GFP to GFP-TRAP-A-based immuno-affinity purification, to isolate intact Augmin (Fig. 1A). Mass spectrometry confirmed 56-84% coverage of each of the 8 Augmin subunits (Msd1, Msd5, Wac, Dgt2-Dgt6), demonstrating the ability of Msd1-GFP to co-precipitate all other Augmin subunits (Table 1). All subunits of Augmin, apart from Msd1, were quantified at approximately equal abundance (Fig. 1B). The presence of approximately three-fold greater Msd1 is likely a consequence of its role as ‘bait’ protein in this methodology, as sucrose gradient density centrifugation of Msd1-GFP extracts demonstrated two populations of Msd1-GFP of sizes corresponding to monomeric and Augmin-incorporated (not shown). Thus, in agreement with previous qualitative observations (Goshima et al., 2008), *Drosophila* Augmin possesses a subunit stoichiometry of 1:1.

**Table 1.**
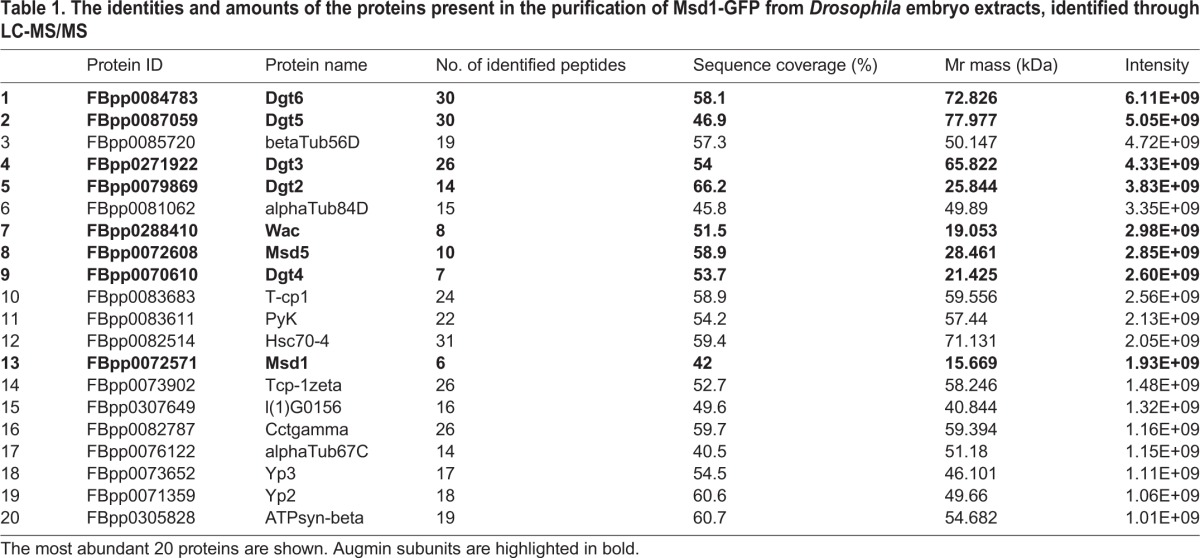
**The identities and amounts of the proteins present in the purification of Msd1-GFP from *Drosophila* embryo extracts, identified through LC-MS/MS**

To obtain structural information on the relationship between Augmin subunits, we subjected purified Augmin on beads to chemical cross-linking using bis[sulfosuccinimidyl] suberate (BS3), followed by trypsin digestion and mass spectrometry (see the Materials and Methods). We then identified cross-linked peptides between and within Augmin subunits. No cross-links were identified between Augmin proteins and proteins co-purified on GFP-TRAP-A beads, suggesting that these additional proteins bind non-specifically to the GFP-TRAP-A beads, rather than being Msd1-GFP/Augmin interacting proteins (not shown).

Our IP-CLMS analysis identified 77 intra-protein linkages, and 59 inter-protein linkages within Augmin at 5% FDR (Table 2). A predicted molecular topology of *Drosophila* Augmin was constructed from this data, revealing a set of potential inter-connections between the eight Augmin subunits, where seven subunits interact with two or more others (Fig. 1C). The structural restraints suggest a ‘core’ of interactions centred around the C-termini of Dgt5, Dgt3 and Wac, the N-terminus of Dgt6 and the Dgt2, Msd1 and Msd5 subunits. Such a complex network of interactions provides a molecular explanation for the reported inter-dependence of these subunits, in terms of Augmin stability (Goshima et al., 2008; Meireles et al., 2009): removal of one of these core subunits could theoretically lead to the complex instability observed *in vivo*. (Fig. 1C). This predicted topology differs in some aspects with the recently proposed *in vitro* reconstituted network of human Augmin subunits (Hsia et al., 2014). In that *in vitro*-driven approach, human Dgt4 (HAUS8, previously known as HICE1) was placed within a central dimer, together with hDgt6 (HAUS6), interacting with the four non-conserved subunits to constitute a hexamer (Hsia et al., 2014). In contrast, our *in vivo*-driven CLMS map suggests Dgt4, with only a single, weak predicted interaction, lies on the outside of core Augmin. However, in both studies, Dgt3/HAUS3 and Dgt5/HAUS5 appear to have structurally distinct properties to the rest of Augmin.

**Table 2.**
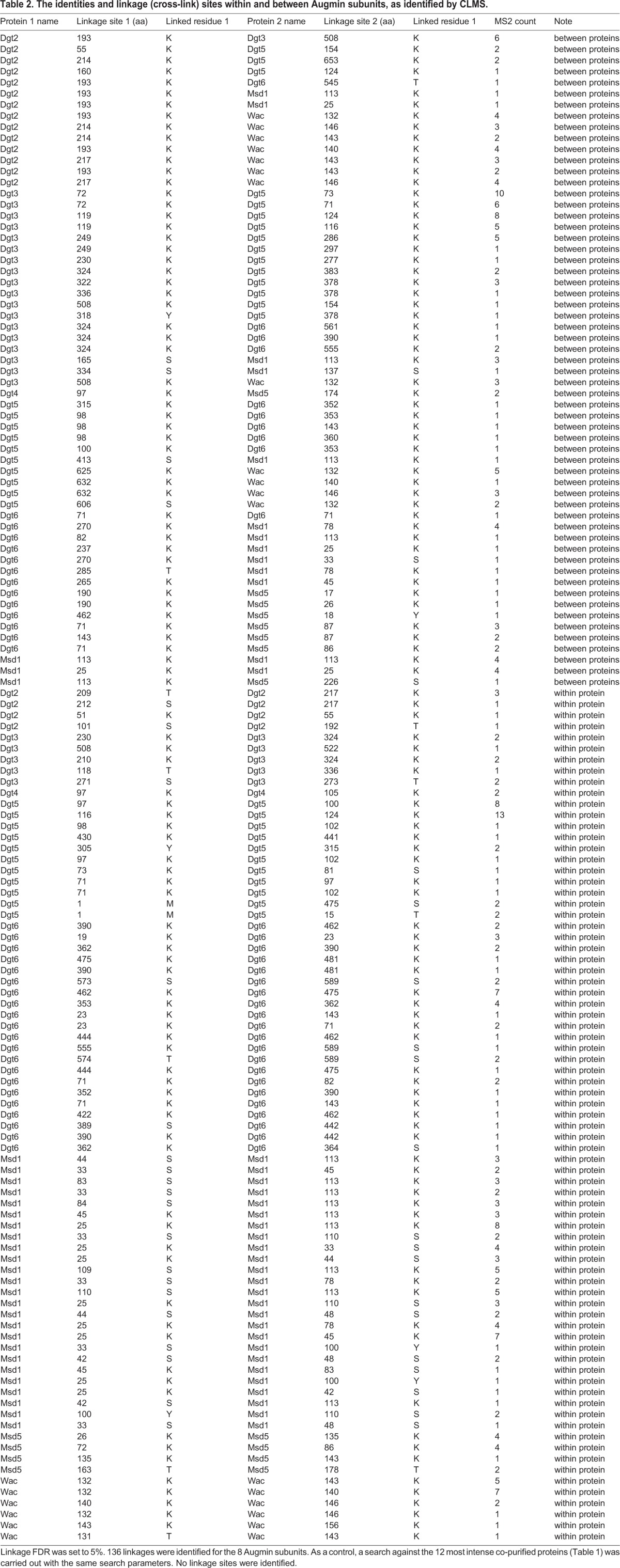
**The identities and linkage (cross-link) sites within and between Augmin subunits, as identified by CLMS.**

Our analysis also identified 10 parallel cross-links along the length of the N-termini of Dgt3 and Dgt5 (∼aa 75-350), suggesting the possibility of a hetero-dimeric sub-complex along this interface. Moreover, an additional six interactions were identified between Dgt6 and Dgt3/5. As the human homologue of Dgt6 (HAUS6) has previously been shown to interact with the NEDD1 subunit of γ-TuRC, we hypothesised that these regions of Dgt3 and Dgt5 might function co-operatively with Dgt6 to recruit *Drosophila* γ-TuRC through the NEDD1 homologue, Dgp71WD.

**Fig. 2.**
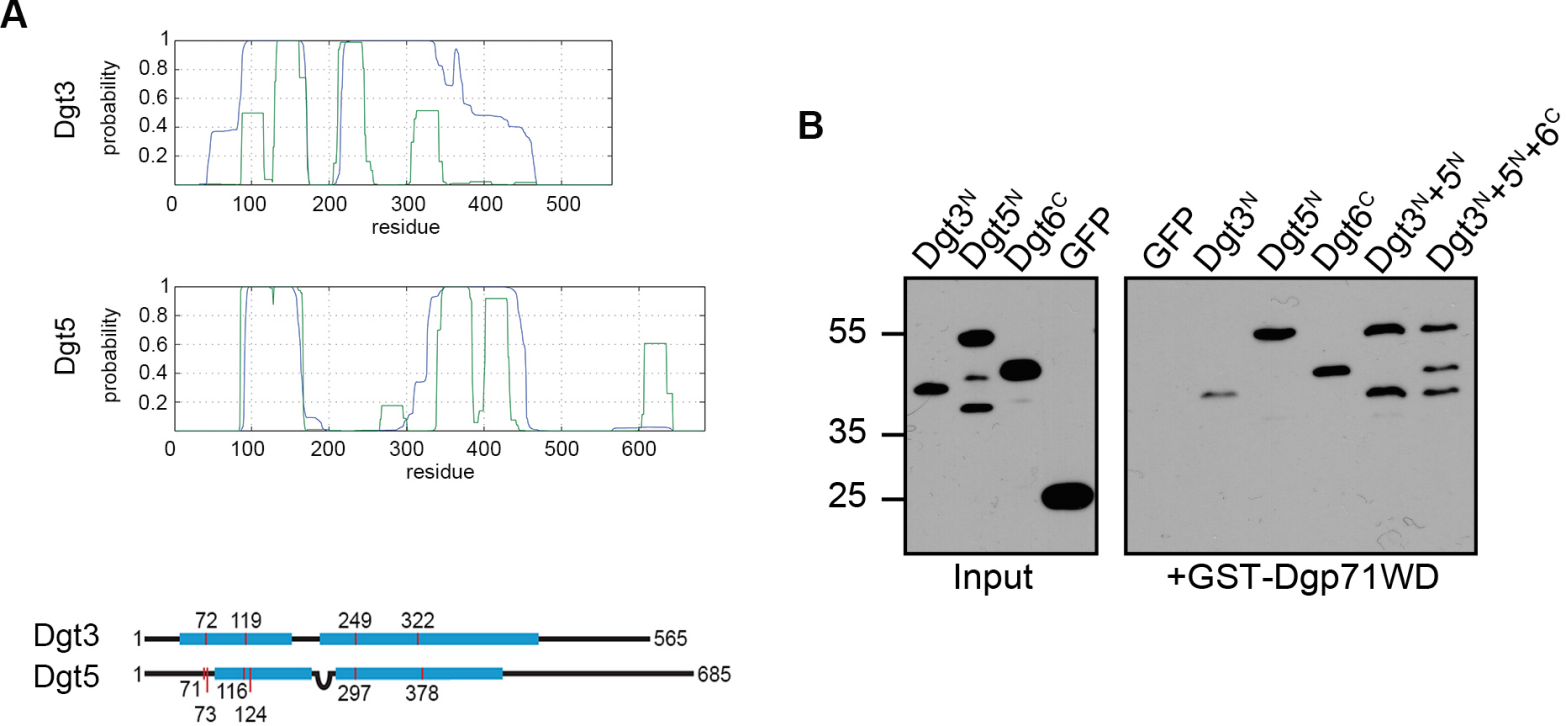
**Bioinformatic and *in vitro* validation of the relationships between Dgt3, Dgt5, Dgt6 and the γ-TuRC subunit, Dgp71WD.** (A) Top two panels show potential coiled coil formation of Dgt3 and Dgt5 as predicted by Multicoil2 (blue) and Marcoil (green) (see Materials and Methods). Both proteins are likely to form two separate segments of homo-dimeric coiled coils. Bottom panel shows Dgt3 and Dgt5 and alignment of the cross-links identified by CLMS. Coiled-coil segments are coloured in cyan, and cross-linked residues in red. Imposition of a short ‘loop’ between the two coiled-coil regions in Dgt5 bring all positional restrictions between Dgt3 and Dgt5 into alignment, strengthening the hypothesis that these proteins form a hetero-dimeric coiled coil. (B) Purified GST-Dgp71WD on glutathione beads incubated with His-Dgt3^N^, His-Dgt5^N^, His-Dgt6^C^ and His-GFP, singly and in combination. His-Dgt5^N^ and His-Dgt6^C^ associate strongly with GST-Dgp71WD. His-Dgt3^N^ associates weakly when incubated singly, but increases affinity in the presence of His-Dgt5^N^. His-GFP provides a negative control and does not associate with GST-Dgp71WD.

Initially, to test this hypothesis, we subjected the N-terminal sequences of Dgt3 and Dgt5 to *de novo* structural bioinformatics predictions (Fig. 2A). This was consistent with a model in which the N-termini of Dgt3 and Dgt5 form coiled coils; when the structural restrictions from our cross-linking experiment are applied, a hetero-dimeric parallel combination was found along two extended regions covering most of the ∼300 length of the interacting polypeptides (Fig. 2A). In contrast, the C-terminus of Dgt6 (∼aa 300-654) is predicted to be disordered, with no structural homology to known protein folds (not shown). We next bacterially expressed and purified His-tagged versions of the N-terminal half of Dgt3 (aa 1-350), Dgt5 (aa 1-450), and the C-terminal portion of Dgt6 (aa 298-654) and tested their ability to interact with a GST-tagged variant of the γ-TuRC subunit, Dgp71WD (Reschen et al., 2012). While GST-Dgp71WD did not interact with a control His-tagged protein (GFP), it was able to sequester all three Augmin polypeptides (Fig. 2B; data not shown). Interestingly, Dgt3^N^ interacted only weakly with GST-Dgp71WD on its own but consistently showed greater affinity in the presence of Dgt5^N^, further supporting the notion that the N-termini of Dgt3 and Dgt5 act co-operatively *in vivo* (Fig. 2B).

**Fig. 3.**
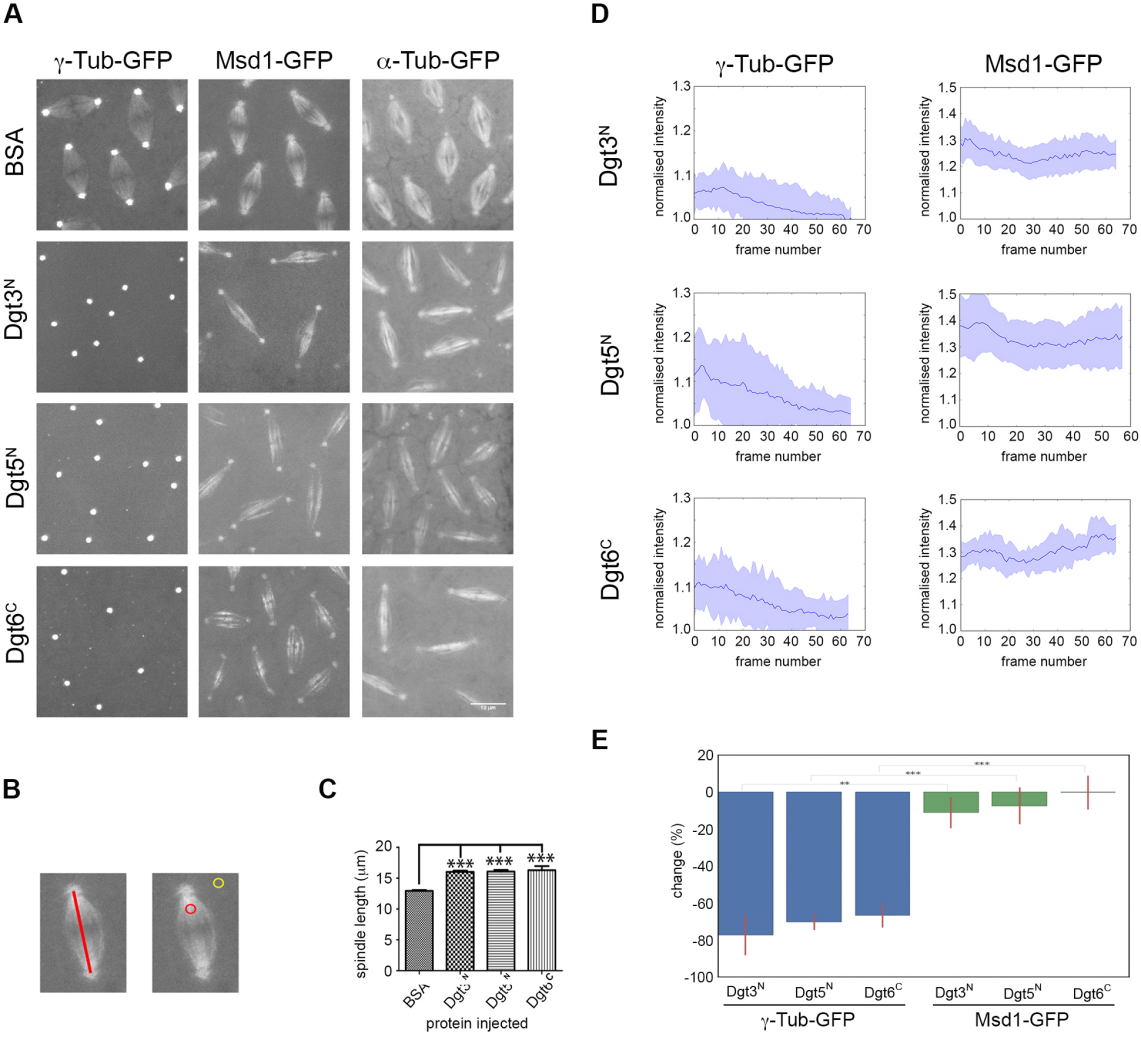
**Dgt3^N^, Dgt5^N^, and Dgt6^C^ are all required to correctly localize γ-Tubulin to the mitotic spindle.** (A) Injections of Dgt3^N^, Dgt5^N^ and Dgt6^C^ into embryos expressing γ-Tubulin-GFP, Msd1-GFP, or Tubulin-GFP. In control injections with BSA, γ-Tubulin-GFP accumulates on the spindle region. In Dgt3^N^, Dgt5^N^, or Dgt6^C^ injections, γ-Tubulin-GFP disappears from the spindle region and embryos arrest with long, thin spindles, as expected for disruption of Augmin function. In both control BSA injections and in Dgt3^N^, Dgt5^N^, or Dgt6^C^ injections, Msd1-GFP localisation reflects the morphology of Tubulin-GFP, demonstrating spindle localisation of Augmin is unaffected. (B) Fluorescence intensity was measured for an area within the spindle (red circle), and an area in the nearby background (yellow circle). Each fluorescence measured for the spindle was normalised to the associated background (see Materials and Methods); spindle length was determined from the distance between the two centrosomes (red line). (C) Cycle 10 spindles are significantly longer when injected with Dgt3^N^, Dgt5^N^, or Dgt6^C^ compared with BSA injection (*P*<0.0001). For each injection, between 24 and 47 spindles were selected from 3-6 embryos for measurement. Error bars show the s.e.m. (D) Graphs showing the fluorescence intensity in the spindle region, normalised to background, over time, following injection of Dgt3^N^, Dgt5^N^, or Dgt6^C^. The line shows the mean, while the coloured area shows the variance between individual spindles (between 10 and 16 spindles, measured from 3 embryos per condition). After Dgt3^N^ injection, level of γ-Tubulin-GFP in the spindle region gradually decreases to near background levels, whereas Msd1-GFP levels stays relatively constant. (E) Bar chart showing the relative fluorescence decrease in spindle fluorescence between initial measurement following injection and *t*=600 s. A two-tailed Mann–Whitney U test confirms that spindle fluorescence of γ-Tubulin-GFP significantly decreases from *t*=0 to *t*=600 s, while Msd1-GFP fluorescence does not. **P<0.001; ***P<0.0001.

To functionally validate the hypothesis that these regions of Dgt3 and Dgt5 have a role in recruiting γ-TuRC to pre-existing MTs, in addition to Dgt6, we injected bacterially expressed, MBP-tagged purified Dgt3^N^, Dgt5^N^ and Dgt6^C^ into *Drosophila* syncytial embryos expressing GFP transgenes. Injection of 5 mg/ml BSA, or the Augmin subunit Wac, had no effect on mitotic progression, spindle architecture or the spindle localisation of either Msd1-GFP or γ-Tubulin-GFP (Fig. 3A; Movies 1-5). However, injection of any of the three truncated proteins following nuclear envelope breakdown resulted in an *augmin*-like phenotype of long, weak density spindles, which arrested at the metaphase/anaphase transition (Fig. 3A-C; Movies 6-8; Wainman et al., 2009; Hayward et al., 2014). We measured the fluorescence intensity of Msd1-GFP on multiple spindles, in multiple embryos for each condition, and found that, in all cases, it did not significantly change over time (Fig. 3D,E; Movies 9-11). Similarly, injection of the truncated proteins into embryos expressing Dgt5-GFP did not result in loss of Dgt5 from spindle MTs (Movies 12-14). This demonstrates that the *augmin*-like phenotype is not a consequence of disrupting the localisation and function of the Augmin complex, per se. In contrast, the intensity of γ-Tubulin-GFP and Dgp71WD on spindles in each condition reduced over time to apparent near-background levels (Movies 15-20). This was quantified for spindle-associated γ-Tubulin-GFP (Fig. 3D). The measured difference in effect between Msd1-GFP and γ-Tubulin-GFP accumulation on the spindle after subunit injection was most apparent, and statistically significant, when the initial fluorescence intensity and the intensity ∼600 s after injection was compared (Fig. 3E). These results therefore support a model in which injected Dgt3^N^, Dgt5^N^ or Dgt6^C^ bind directly to Dgp71WD to sterically interfere with the interaction between endogenous Augmin and γ-TuRC.

Overall, the structural, biochemical and cell biological data presented here suggests a complex mechanism by which *Drosophila* Augmin bridges the gap between pre-existing MTs and γ-TuRC, requiring at least three of the eight subunits. It also demonstrates the power of CLMS as a tool with which to provide testable hypotheses regarding the cellular function of protein complexes for which there is little, or no, structural data; expanding the base of CLMS (Chen et al., 2010; Lasker et al., 2012) to a structure investigation method in its own right. Future investigations of purified Augmin, based on the data here, should shed further light on the precise architecture of Augmin and the mode of action by which it facilitates MT-templated MT nucleation.

## MATERIALS AND METHODS

### GFP-TRAP-A isolation of Augmin

Flies expressing full-length Msd1-GFP via UASp/maternal-α-Tubulin GAL4 control (Wainman et al., 2009) were maintained according to standard procedures at 25°C. Batches of 0- to 3-h-old embryos laid by cages of 1- to10-day-old flies were dechorionated, weighed, flash frozen in N_2_ (l) and stored at −80°C. A total of 8 g of frozen embryos were homogenized in 16 ml C buffer (50 mM HEPES, pH 7.4, 50 mM KCl, 1 mM MgCl_2_, 1 mM EGTA, 0.1% IGEPAL CA-630, Roche protease inhibitors). Extract was clarified through centrifugation at 10,000 ***g*** for 10 min, 100,000 ***g*** for 30 min, and 100,000 ***g*** for a further 10 min. Clarified extract was incubated with 50 μl GFP-TRAP-A beads (Chromotek, Germany) overnight at 4°C to immunoprecipitate Augmin. Msd1-GFP/Augmin-GFP-TRAP-A beads were washed three times with ice-cold C buffer and three times with ice-cold C buffer without IGEPAL CA-630. Based on previous semi-quantitative western blotting (not shown) we estimate 80 μg of Augmin was present in the sample.

### Mass spectrometry sample preparation

To estimate sample quality and digestion efficiency, 2.5% of total beads were analyzed by LC-MS/MS. This Augmin aliquot was resuspended in 50 μl of 50 mM ammonium bicarbonate. Trypsin was added to a final concentration of 20 ng/μl and samples digested at 37°C with shaking overnight. Supernatant (containing peptides) was collected and acetified to pH 3 with 0.1% trifluoroacetic acid. Peptides were subsequently desalted using C18-StageTips (Rappsilber et al., 2003) for mass spectrometric analysis.

The remaining (97.5%) purified Augmin was resuspended in 200 μl C buffer and cross-linked using 400 μg of bis[sulfosuccinimidyl] suberate (BS3) [i.e. 1:5 protein to cross-linker ratio (g/g)]. The cross-linking reaction was incubated on ice for 2 h with periodic agitation. After removal of supernatant, the beads were incubated with 200 μl of 50 mM ammonium bicarbonate for 30 min on ice with periodic agitation; then, 3 μg trypsin was added and digestion left to occur at 37°C with shaking overnight. After digestion, peptide mixture (in supernatant) was collected and fractionated using SCX-StageTips (Rappsilber et al., 2003) with a small variation to the protocol previously described for linear peptides (Ishihama et al., 2006). In short, the peptide mixture was acetified with 2.5% acetic acid to pH3 and was loaded on a SCX-Stage-Tip. The bound peptides were eluted in four steps with buffers (10% v/v ACN, 0.5% v/v acetic acid) containing 50 mM, 100 mM, 200 mM and 500 mM ammonium acetate into four fractions. Cross-linked peptides were expected to be in the three fractions that were eluted with higher ammonium acetate concentrations. Peptides in these three fractions were desalted using C18-StageTips (Rappsilber et al., 2007) prior to mass spectrometric analysis.

### Mass spectrometric analysis

Samples were analyzed using an LTQ-Orbitrap mass spectrometer (ThermoElectron, Germany) in order to determine composition. Peptides were separated on an analytical column packed with C18 material (ReproSil-Pur C18-AQ 3 μm; Dr Maisch GmbH, Ammerbuch-Entringen, Germany) in a spray emitter (75 μm inner diameter, 8 μm opening, 250 mm length; New Objectives). Mobile phase A consisted of water and 0.5% acetic acid. Mobile phase B consisted of acetonitrile and 0.5% acetic acid. Peptides were loaded at a flow rate of 0.5 μl/min and eluted at 0.3 μl/min using a linear gradient going from 1% B to 32% B in 55 min followed by a linear increase from 32% to 76% in 5 min. The eluted peptides were directly introduced into the mass spectrometer. MS data was acquired in the data-dependent mold. For each acquisition cycle, the mass spectrometric spectrum was recorded in the orbi-trap with a resolution of 60,000. The 20 most intense ions in the with a precursor charge state 2+ or higher were fragmented in the ion-trap by collision-induced disassociation. The fragmentation spectra were then recorded in the LTQ linear ion trap at normal scan rate. Dynamic exclusion was enabled with single repeat count and 60 s exclusion duration.

SCX-Stage-Tip fractions were analyzed using same LC-MS/MS system as described above however with a high-high strategy. Peptides were loaded at a flow rate of 0.5 μl/min and eluted at 0.3 μl/min using a linear gradient going from 3% B to 35% B in 130 min followed by a linear increase from 35% to 80% in 5 min. The eluted peptides were directly introduced into the mass spectrometer. MS data was acquired in the data-dependent mold. For each acquisition cycle, the mass spectrometric spectrum was recorded in the orbi-trap with a resolution of 100,000. The eight most intense ions in the with a precursor charge state 3+ or higher were fragmented in the ion-trap by collision-induced disassociation. The fragmentation spectra were then recorded in the orbi-trap at a resolution of 7500. Dynamic exclusion was enabled with single repeat count and 60 s exclusion duration.

### Identification of proteins in the purified Augmin sample

The raw mass spectrometric data of the purified Msd1-GFP/Augmin-GFP-TRAP-A beads sample was processed into peak list using MSCover module from Proteowizard (v.3.0.3414) (Kessner et al., 2008). Database search was conducted using Mascot (v. 2.4) (Matrix Sciences). Specified database search parameters were: MS accuracy, 6 ppm; MS/MS accuracy, 0.5 Da; enzyme, trypsin; variable modification, oxidation on methionine; database, dmel-all-translation-r5.48 database (FlyBase); protein FDR, 1%. Protein abundance in the sample was estimated based on PAI value (Ishihama et al., 2008). The top 20 identified proteins (based on abundance) are listed in Table 1.

### Identification of cross-linked peptides

The raw mass spectrometric data files of SCX fractions of cross-linked Msd1-GFP/Augmin were processed into peak lists using MaxQuant version 1.2.2.5 (Cox and Mann, 2008) with default parameters, except ‘Top MS/MS Peaks per 100 Da’ was set to 20. The peak lists were searched against the sequences of the eight Augmin subunits, using Xi software (ERI, Edinburgh) for identification of cross-linked peptides. Search parameters were as follows: MS accuracy, 6 ppm; MS/MS accuracy, 20 ppm; enzyme, trypsin; specificity, fully tryptic; allowed number of missed cleavages, four; cross-linker, BS3; fixed modifications, carbamidomethylation on cysteine; variable modifications, oxidation on methionine. The linkage specificity for BS3 was assumed to be for lysine, serine, threonine, tyrosine and protein N-termini. Linkage FDR was set to 5%. As a control, a search against the 20 most abundant proteins identified from the purified Msd1-GFP/Augmin-GFP-TRAP samples (including 8 Augmin subunits and the 12 most intense co-purified proteins; Table 1) was carried out with the same search parameters.

### Protein expression and purification

pGEX-Dgp71WD was a gift from Jordan Raff (University of Oxford, UK). pQE80-His-GFP was obtained from Steven Porter (University of Exeter, UK). pRSETA-Dgt3^N^, pRSETA-Dgt5^N^, and pRSETA-Dgt6^C^ were created using the GeneArt service (Life Technologies). pRSETA-Dgt3^N^ constituted aa 1-350 of Dgt3-PA, pRSETA-Dgt5^N^ constituted aa 1-450 of Dgt5-PA and pRSETA-Dgt6^C^ constituted aa 298-654 of Dgt6-PA.

All plasmids were transformed into BL21 (DE3) cells and grown in LB medium at 37°C to an OD_600_ of between 0.4-0.6 before induction with 0.1 mM IPTG. pGEX-Dgp71WD and pQE80-His-GFP were induced at 18°C overnight, pRSETA-Dgt3^N^, pRSETA-Dgt5^N^, and pRSETA-Dgt6^C^ were induced at 4°C overnight. Cells were pelleted at 6800 g and stored at −80°C until required.

Bacteria expressing GST-Dgp71WD were incubated in Buffer A (PBS adjusted to 900 mM NaCl, 0.5% IGEPAL CA-630, 0.2 mg/ml lysozyme, 1 mM PMSF) for 1 h with rotation at 4°C, sonicated with 6×10 s bursts and centrifuged at 10,000 ***g*** for 10 min at 4°C to pellet cell debris. The supernatant was incubated with Glutathione Agarose beads (Sigma) overnight at 4°C with rotation and washed twice with 10 volumes of Buffer A and once with PBS, ready for use in the GST-pull down assay (see following section). Bacteria expressing His-tagged Dgt3^N^, Dgt5^N^, Dgt6^C^ were incubated in Buffer B (PBS adjusted to 500 mM NaCl, 0.5% IGEPAL CA-630, 10 mM imidazole, 0.2 mg/ml lysozyme, 1 mM PMSF) for 1 h with rotation at 4°C, sonicated with 6×10 s bursts and centrifuged at 10,000 ***g*** for 10 min at 4°C to pellet cell debris. The supernatant was incubated with HisPur Cobalt resin (Pierce) for 2 h with rotation at 4°C, before being loaded into a standard 1 ml column (Pierce), washed with at least 20 volumes of Buffer B and eluted with PBS containing 150 mM imidazole, and 0.1% IGEPAL CA-630. His-tagged GFP was purified as above, except using Buffer D (PBS adjusted to 500 mM NaCl, 25 mM imidazole) and Ni-Sepharose Fast Flow resin (GE). His-tagged proteins were concentrated using an Amicon Ultra column (30 kDa cut-off) and buffer exchanged into Buffer A, for immediate use in the GST-pull down assay.

### GST-pull down assay

GST and GST-Dgp71WD, immobilized on Glutathione Agarose resin, was washed three times with PBS containing 150 mM imidazole, and 0.1% IGEPAL CA-630. Approximately 20 µg of His-tagged protein were incubated with 10 µl of resin for 2 h at 4°C with agitation. After incubation, resins were washed three times with PBS containing 150 mM imidazole, and 0.1% IGEPAL CA-630 and re-suspended in 30 µl of protein sample buffer for SDS-PAGE/western blotting analysis.

### SDS PAGE and Western blotting

Samples were subjected to standard SDS-PAGE and Western blotting. Membranes were blocked with 0.1% PBS-T+5% milk for 1 h at RT prior to incubation with primary antibodies. His-Tag (27E8) mouse monoclonal antibody (Cell Signaling Technology, 2366) was used at 1:1000.

### Bioinformatics

Potential coiled-coil formation was assessed by two independent algorithms: Multicoil2 (a modern algorithm that combines probabilistic sliding window method with Hidden Markov Model (HMM) approaches) (Trigg et al., 2011) and Marcoil (based on window-less HMMs, optimised for the simultaneous recognition of domains of different lengths) (Delorenzi and Speed, 2002).

To assess if the Dgt3-Dgt5 potential hetero-dimeric coiled coil has a preferred register, we used MODELLER (Šali and Blundell, 1993) to generate models of coiled coils based on Liprin-beta-2 structure (PDB ID: 3QH9). While each molecule was forced into a continuous alpha-helix, the interatomic distances in one chain were constrained to be identical with the other, creating a symmetrical hetero-dimeric parallel coiled coil. We calculated a number of models that differed in heptad-repeat register between Dgt3 and Dgt5. Although cross-linked lysines 71 and 73 of Dgt5 lay outside of the predicted coiled coil, we extended our models to include these residues, to assess if a long stretch of coiled coil could impose its super-secondary structural fold on neighboring residues. We define position ‘0’ as a heptad-repeat match between Dgt3 and Dgt5, where residue number difference between any two corresponding positions in the repeat is minimal. Position ‘+1’ refers to a slide by 1 heptad-repeat of Dgt5 towards its N-terminus, and ‘−1’ towards its C-terminus. To evaluate which model simulates the experimental data better, we used program Xwalk (Kahraman et al., 2011) to calculate distances between beta-carbons of cross-linked lysines (Euclidean distance). Since such distance vectors can penetrate the surface of the protein, Xwalk also computes the shortest path between the two cross-linked lysines by following the solvent accessible protein surface distance (SASD).

Potential of Augmin subunits to be disordered was assessed by two algorithms: DISOPRED2 (Ward et al., 2004) and PrDOS (Ishida and Kinoshita, 2007). PrDOS judges disorder by local amino acid sequence (sliding window), using support vector machine learning, and also by template prediction based on conservation of disorder in related protein families (subject to availability of high-resolution structural data). DISOPRED2 identifies disordered residues in a similar fashion – based on prior knowledge of crystal structures (missing residues in electron density), and also through a local sequence profile classification using neural networks (sliding window).

### Drosophila stocks

The Msd1-GFP flies have been previously reported (Wainman et al., 2009). To follow Dgt5 localisation *in vivo*, full-length *dgt5* was cloned into the Gateway expression vector pPWG (*Drosophila* Genome Resource Center) via pENTR/D/TOPO. The plasmid was injected into w^1118^ embryos by BestGene, Inc. In both cases, expression was driven in the female germline using the Maternal-α-Tubulin VP16GAL4 line (Bloomington Stock Center, Indiana University, USA). Flies expressing α-Tubulin-GFP were obtained from the Bloomington Stock Center. Flies expressing γ-Tubulin-GFP, under the control of the Ncd promoter, were a gift from Sharyn Endow. Flies expressing Dgp71WD under the control of the Polyubiquitin promoter were a gift from Jordan Raff (University of Oxford, UK).

### *Drosophila* embryo microinjections and imaging

*Drosophila* embryos 1- to 2-h-old were harvested from 1- to 5-day-old adults. Embryos were manually dechorionated and mounted on 22×50 cm coverslips with heptane glue. The embryos were covered with 1:1 mixture of halocarbon oil 700 and halocarbon oil 27 (Sigma). Images were acquired with Visitron Systems Olympus IX81 microscope with a CSO-X1 spinning disk using a UPlanS APO 1.3 NA (Olympus) 60× objective. Images were acquired at 10 s intervals, in which five stacks 1 µm apart were taken with 400 ms exposure with 10% laser power for all genotypes, except embryos expressing Dgp71WD-GFP, where 20% laser power was used. His-tagged Wac, Dgt3^N^, Dgt5^N^, and Dgt6^C^ were buffer-exchanged with injection buffer (100 mM HEPES pH 7.4, 50 mM KCl) and concentrated with 30 kDa size-exclusion columns (Amicon). Protein concentration was measured by Bradford assay. Proteins were injected at 5 µg/µl. Embryos were injected using Eppendorf Inject Man NI 2 and Femtotips II needles (Eppendorf).

### Image analysis

Image processing and analysis were undertaken using Fiji software. The five stacks taken for each time-frame were combined under maximum projection. For each of the BSA, Dgt3^N^, Dgt5^N^, and Dgt6^C^ injections, 3-6 embryos at cycle 10 and between 24 and 47 spindles were selected for length measurements. The distances from the centrosome pairs were measured when the metaphase spindles reached a maximum length, determined visually (Fig. 3C). For each of the Dgt3^N^, Dgt5^N^, and Dgt6^C^ injections, three embryos expressing γ-Tubulin-GFP, and three embryos expressing Msd1-GFP were selected for fluorescence intensity measurements, as follows: Photobleaching was accounted for using the ratio method as described in (Phair et al., 2003) and used in the widely-used ImageJ bleach correction plug in (Miura et al., 2014). Each spindle intensity measurement was normalised by dividing by a nearby background value. The resulting value was converted to a percent of maximum above background so that the relative fluorescence decrease between γ-Tubulin-GFP and Msd1-GFP could be compared. A significant decrease of fluorescence intensity was determined by comparing the values between γ-Tubulin-GFP and Msd1-GFP 600 s post initial measurement. A two-tailed Mann–Whitney U test was performed on the distributions of the resulting percentage changes using the Scipy Python library (Jones et al., 2001) to produce *P* values for significance levels as indicated in Fig. 3.
